# Author Correction: Universality of defect-skyrmion interaction profiles

**DOI:** 10.1038/s41467-019-09202-0

**Published:** 2019-03-15

**Authors:** Imara Lima Fernandes, Juba Bouaziz, Stefan Blügel, Samir Lounis

**Affiliations:** Peter Grünberg Institut and Institute for Advanced Simulation, Forschungszentrum Jülich and JARA, 52425 Jülich, Germany

Correction to: *Nature Communications* 10.1038/s41467-018-06827-5; published online: 22 October 2018

The original version of this Article contained an error in the Acknowledgements, which incorrectly omitted from the end the following: ‘We acknowledge the computing time granted by the JARA-HPC Vergabegremium and VSR commission on the supercomputer JURECA at Forschungszentrum Jülich.’ This has been corrected in both the PDF and HTML versions of the Article.

