# Supplementary material for: Universality of defect-skyrmion interaction profiles
Source: Nat Commun. 2018 Oct 22;9:4395. doi: 10.1038/s41467-018-06827-5 (PMC6197213; doi:10.1038/s41467-018-06827-5)
Supplement: Supplementary file 1 — Supplementary Information [file 41467_2018_6827_MOESM1_ESM.pdf]

Universality of defect-skyrmion interaction profiles

Fernandes et al.

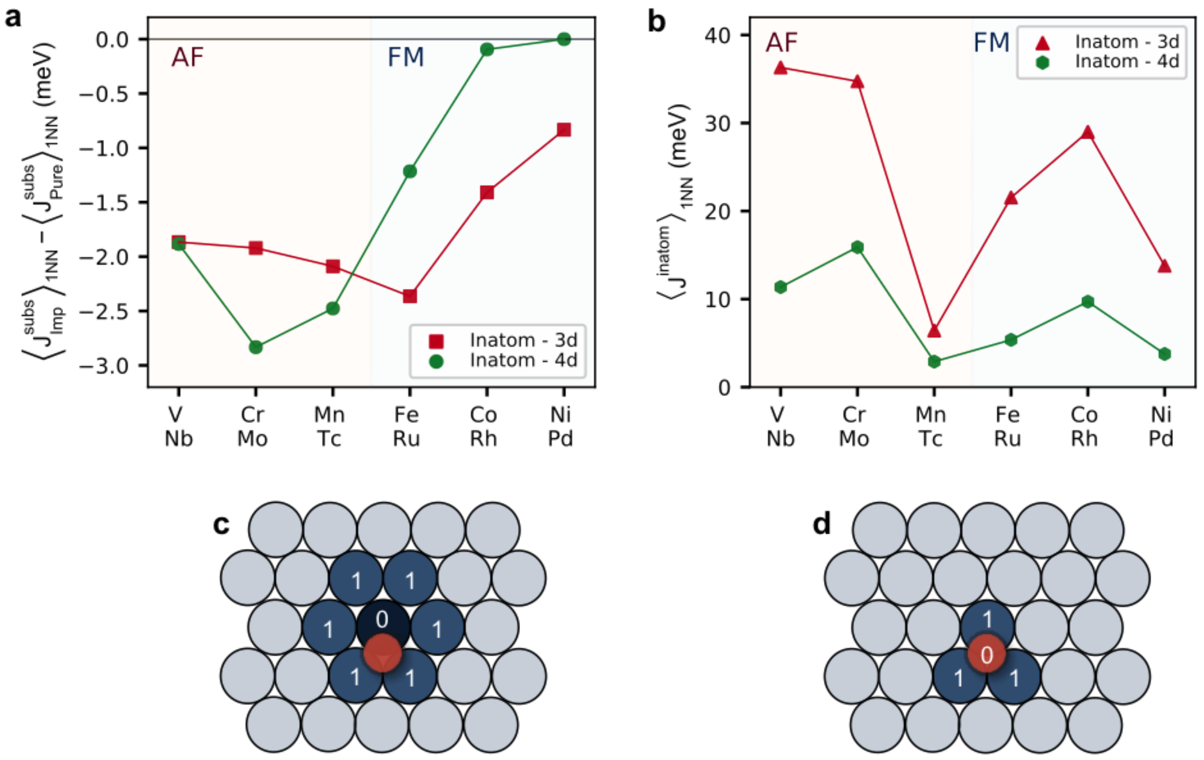

Supplementary Figure 1: Impact of an inatom on the magnetic exchange interaction. (**a**) Difference on the Fe-Fe magnetic exchange interaction for the nearest neighbor with a 3d-inatom (red curve) or 4d-inatom (green curve) and the one of the pure system as a function of the atomic number. (**b**) Absolute value of the inatom-Fe magnetic exchange interaction as a function od the atomic number. The figure (**a**) and (**b**) are divided into two regions indicating the magnetic coupling of the defects with the Fe substrate: antiferromagnetic (AF) on the left and ferromagnetic (FM) on the right. (**c-d**) Illustrative top view of the Fe layer (blue spheres) with the defect position (red sphere). The Fe atoms surrounding the defects are not equivalent, thus we are performing an average of the magnetic exchange interactions over the first neighbor shell.

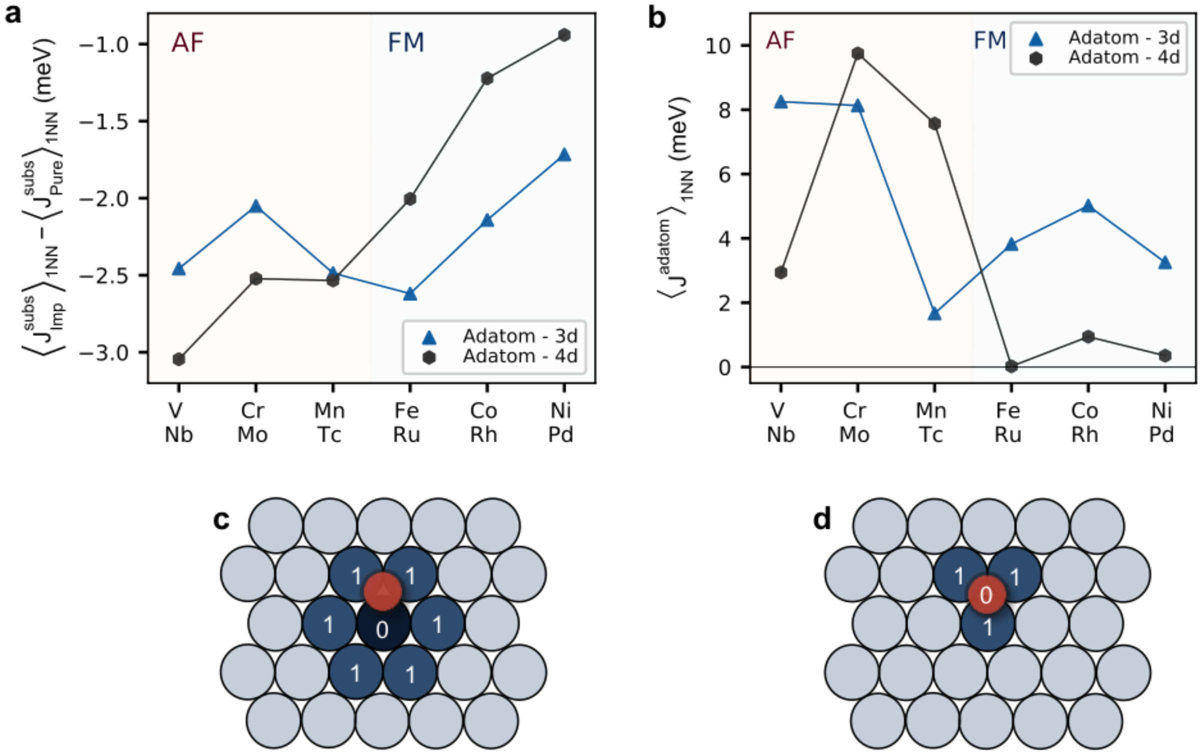

Supplementary Figure 2: Impact of an adatom on the magnetic exchange interaction. (**a**) Difference on the Fe-Fe magnetic exchange interaction for the nearest neighbor with a 3d-inatom (blue curve) or 4d-inatom (gray curve) and the one of the pure system as a function of the atomic number. (**b**) Absolute value of the inatom-Fe magnetic exchange interaction as a function od the atomic number. The figure (a) and (b) are divided into two regions indicating the magnetic coupling of the defects with the Fe substrate: antiferromagnetic (AF) on the left and ferromagnetic (FM) on the right. (**c-d**) Illustrative top view of the Fe layer (blue spheres) with the defect position (red sphere). The Fe atoms surrounding the defects are not equivalent, thus we are performing an average of the magnetic exchange interactions over the first neighbor shell.

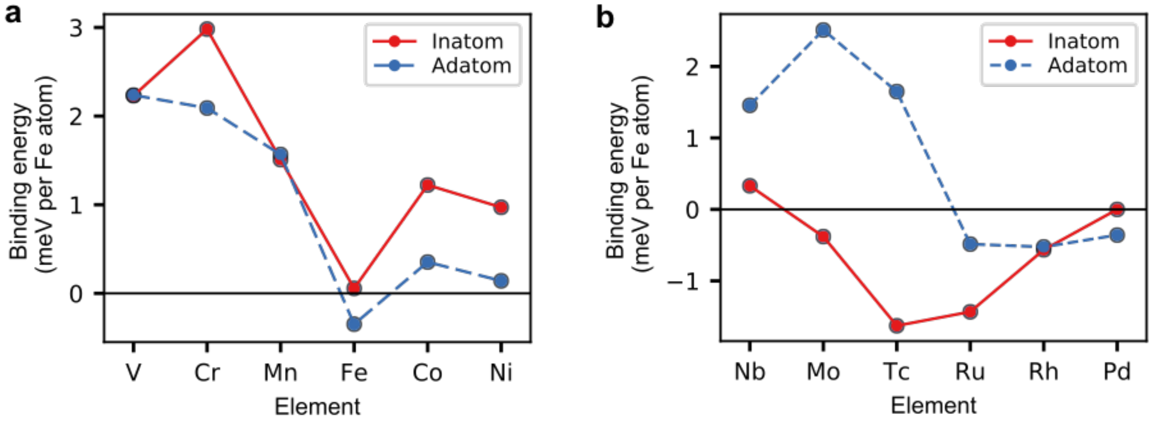

Supplementary Figure 3: Skyrmion-defect interaction within the Heisenberg model. Impact of band-filling on the binding energies considering inatoms (solid) and adatoms (dashed) located close to the Skyrmion center for the (**a**) 3d-series and (**b**) 4d-series. The energies were calculated within the Heisenberg Model.

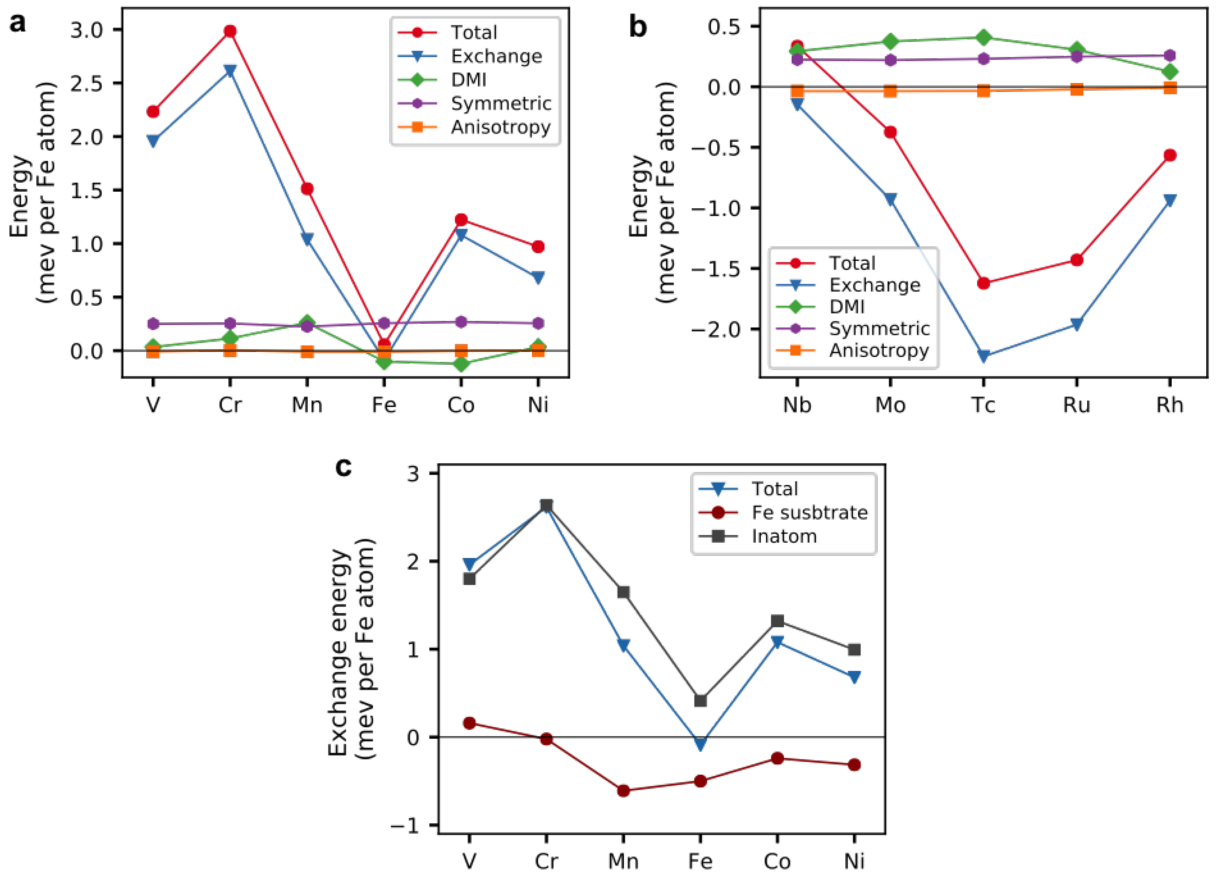

Supplementary Figure 4: Skyrmion-defect binding energies contribution. The total binding energy (red lines) is decomposed into the contribution from the exchange interaction (blue line), the Dzyaloshinskii-Moriya interaction (green line), symmetric interaction (purple line) and anisotropy interaction (orange line) as a function of the atomic number for the (**a**) 3d inatom and (**b**) 4d-inatom. (**c**) The total exchange energy (blue line) is decomposed into the contribution from Fe substrate (gray lines) and from the inatom (gray lines).

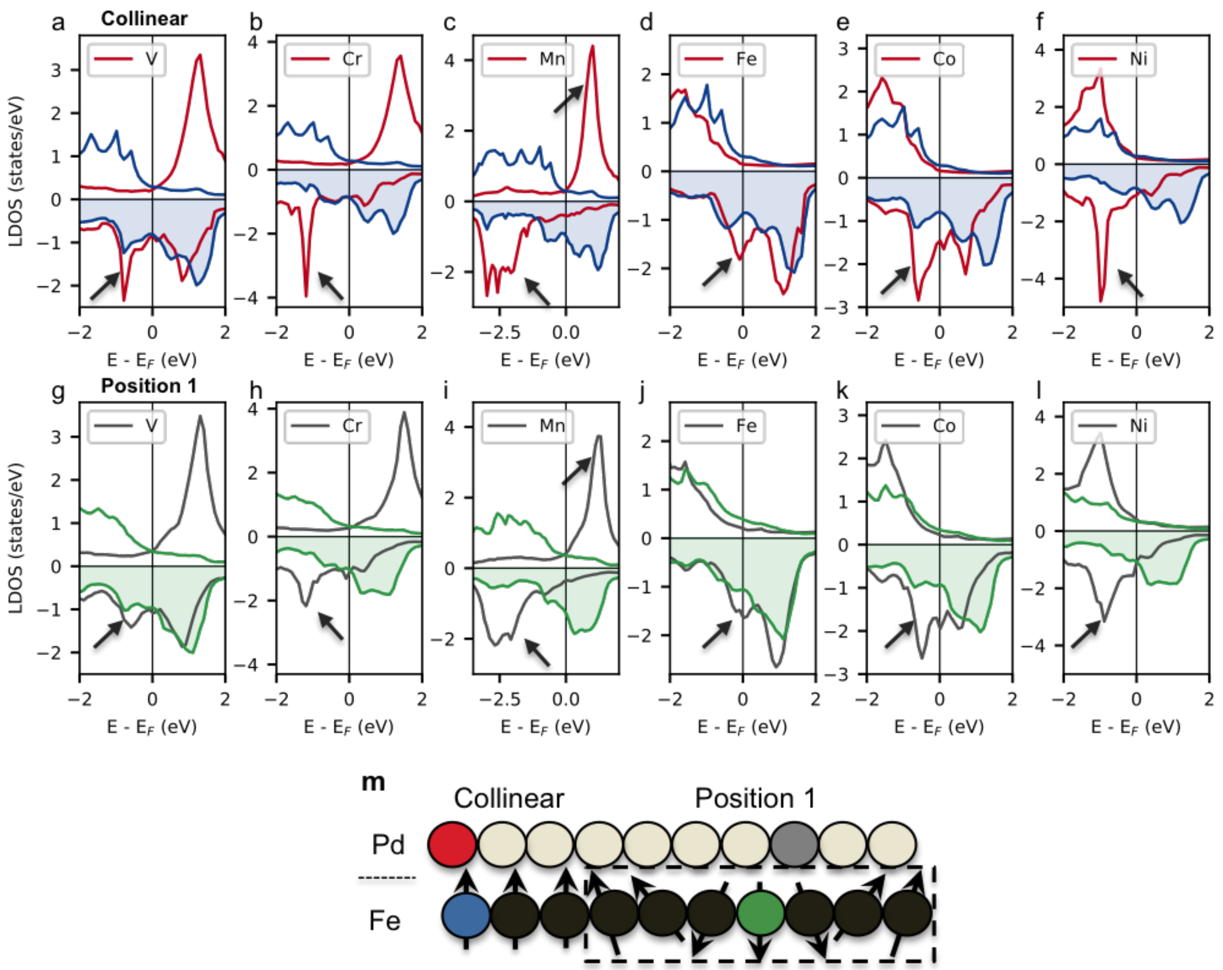

Supplementary Figure 5: Impact of magnetic environment on the impurities electronic structure of the 3d-inatom series. (**a-l**) The local density of states (LDOS) of 3d-inatom series is shown within the collinear region (red lines in **a-f**) or close to the skyrmion core (grey lines in **g-l**). For comparison, the LDOS of one of the closest Fe atoms is plotted at the ferromagnetic background (blue lines in **a-f**) and in the skyrmion-case (green lines in **g-l**). (**e**) Illustrative legend for the colors used.

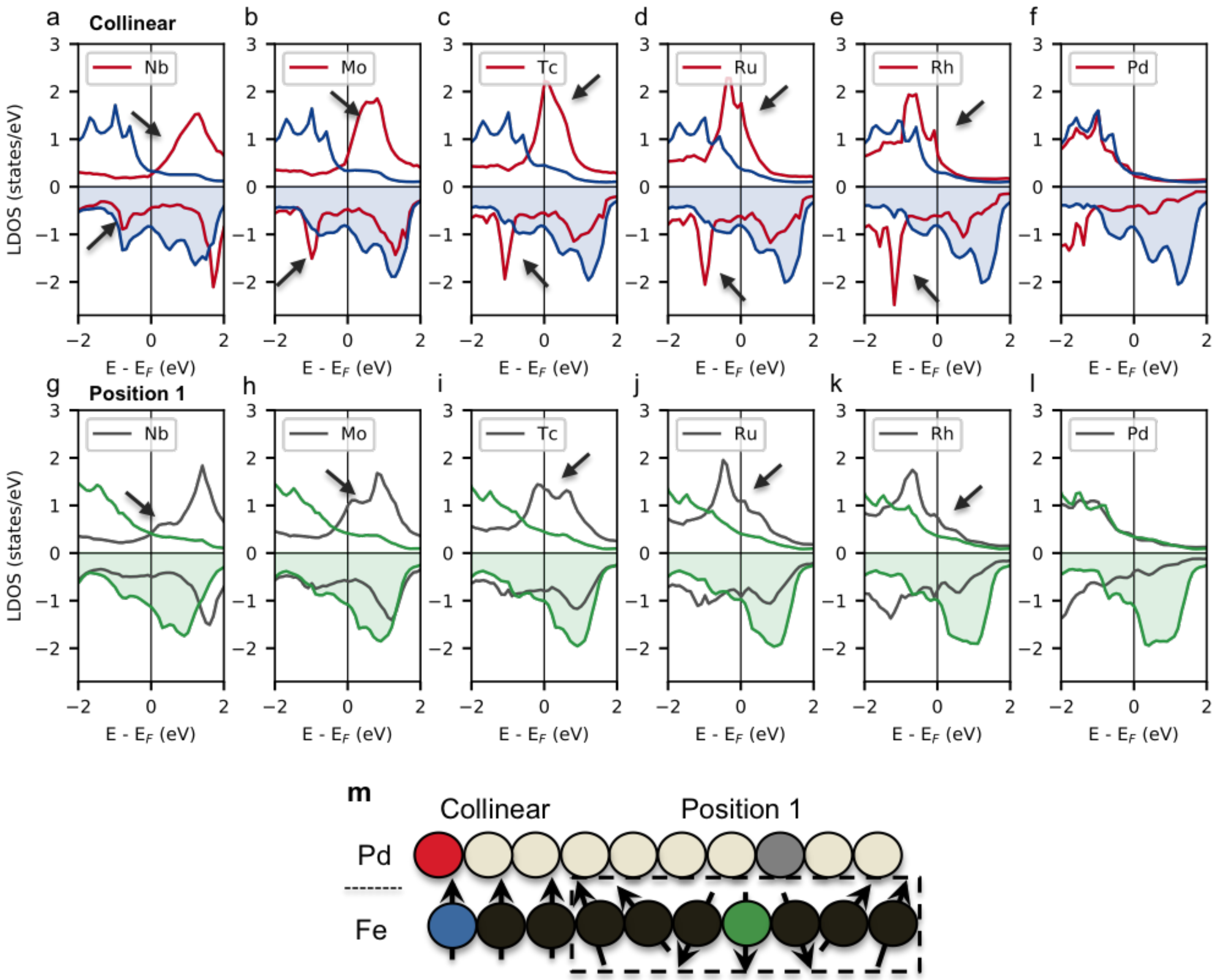

Supplementary Figure 6: Impact of magnetic environment on the impurities electronic structure of the 4d-inatom series (**a-l**) The local density of states (LDOS) of 4d-inatom series is shown within the collinear region (red lines in **a-f**) or close to the skyrmion core (grey lines in **g-l**). For comparison, the LDOS of one of the closest Fe atoms is plotted at the ferromagnetic background (blue lines in **a-f**)and and in the skyrmion-case (green lines in **g-l**). (**e**) Illustrative legend for the colors used.

		V	Cr	Mn	Fe	Co	Ni
Inatom	$\left\langle J_{\text{imp}}^{\text{subs}} \right\rangle_{\text{1NN}}$	14.5	14.5	14.3	14.0	15.0	15.6
	$\left\langle \widetilde{J}_{\text{imp}}^{\text{subs}} \right\rangle_{\text{1NN}}$	16.0	16.1	15.9	15.6	16.6	17.3
	K^{imp}	-0.6	-0.4	-0.5	-0.3	-0.1	0.1
Adatom	$\left\langle J_{\text{imp}}^{\text{subs}} \right\rangle_{\text{1NN}}$	14.1	14.5	14.1	14.0	14.6	14.9
	$\left\langle \widetilde{J}_{\text{imp}}^{\text{subs}} \right\rangle_{\text{1NN}}$	16.4	17.0	16.9	16.1	16.7	17.3
	K^{imp}	0.6	0.9	1.2	0.6	-0.5	0.4

Supplementary Table 1: Heisenberg parameters for 3d-inatoms and 3d-adatoms. The exchange interaction isotropic coupling among the Fe atom and the first-nearest neighbors $\left(\left\langle J_{\text{imp}}^{\text{subs}} \right\rangle_{\text{1NN}}\right)$ and the renormalized magnetic exchange for the Fe substrate $\left(\left\langle \widetilde{J}_{\text{imp}}^{\text{subs}} \right\rangle_{\text{1NN}}\right)$ and the magnetic anisotropy energy of the inatom defect $\left(K^{\text{imp}}\right)$ are given in meV.

		Nb	Mo	Tc	Ru	Rh	Pd
Inatom	$\left\langle J_{\text{imp}}^{\text{subs}} \right\rangle_{\text{1NN}}$	14.6	13.6	13.9	15.2	16.3	16.4
	$\left\langle \widetilde{J}_{\text{imp}}^{\text{subs}} \right\rangle_{\text{1NN}}$	14.8	13.9	15.0	17.4	18.9	19.0
Adatom	$\left\langle J_{\text{imp}}^{\text{subs}} \right\rangle_{\text{1NN}}$	14.6	13.6	13.9	15.2	16.3	16.4
	$\left\langle \widetilde{J}_{\text{imp}}^{\text{subs}} \right\rangle_{\text{1NN}}$	15.8	17.1	15.6	16.8	17.6	18.1
	K^{imp}	2.7	0.7	3.8	-	-	-

Supplementary Table 2: Heisenberg parameters for 4d-inatoms and 4d-adatoms. The exchange interaction isotropic coupling among the Fe atom and the first-nearest neighbors $\left(\left\langle J_{\text{imp}}^{\text{subs}} \right\rangle_{\text{1NN}}\right)$ and the renormalized magnetic exchange for the Fe substrate $\left(\left\langle \widetilde{J}_{\text{imp}}^{\text{subs}} \right\rangle_{\text{1NN}}\right)$ and the magnetic anisotropy energy of the inatom defect $\left(K^{\text{imp}}\right)$ are given in meV.

Supplementary Note 1 − Heisenberg model

Details of the model To study the influence of the different magnetic interactions on the stability of magnetic skyrmions in PdFe/Ir(111) at the vicinity of a single atomic defect, we describe the magnetic moments in terms of a classical spin model and employ the extended Heisenberg model:

$$H = -\sum_{ij} \mathbf{m}_i \, \mathcal{J}_{ij} \, \mathbf{m}_j + \sum_i K \left(\mathbf{m}_i^z\right)^2 , \tag{1}$$

where the unit vectors $\mathbf{m}_i = \mathbf{M}_i/M_i$ define the direction of the atomic magnetic moment $\mathbf{M}_i$ at site i. The first term in Supplementary Equation 1 describes the magnetic interactions between the magnetic moments via the tensorial exchange coupling $\mathcal{J}_{ij}$ extracted from the Full-potential relativistic Korringa-Kohn-Rostoker Green function (KKR) method using the infinitesimal rotation method [1–3]. The second term represents the magnetocrystalline anisotropy where $K > 0$ indicates a preferred in-plane orientation of the magnetic moment.

The tensorial exchange coupling can be separated into three parts

$$\sum_{ij} J_{ij} \left(\mathbf{m}_i \cdot \mathbf{m}_j\right) + \sum_{ij} \mathbf{D}_{ij} \cdot \left(\mathbf{m}_i \times \mathbf{m}_j\right) + \sum_{ij} \mathbf{m}_i \, \mathcal{J}_{ij}^s \, \mathbf{m}_j,$$

where the first term is the Heisenberg exchange interactions. Throughout this supplementary material, a negative (positive) J_{ij} describes an anti-ferromagnetic (ferromagnetic) coupling. The second term is an asymmetric component of the tensor and corresponds to the Dzyaloshinskii-Moriya (DM) interaction which is characterized by the DM vector $\mathbf{D}_{ij}$. The last term represents the traceless symmetric part of the exchange tensor.

Magnitude of exchange interactions and band filling For the pure system, the Fe-Fe nearest-neighbor isotropic exchange interaction ($J^{\mathrm{subs}}_{\mathrm{Pure}}$ = 16.4 meV) and the DM interaction ($D^{\mathrm{subs}}_{\mathrm{Pure}} = 1.2$ meV) are in good agreement with available data utilizing other methods (e.g. 14.7 meV and 1.0 meV in Ref.[4]). The presence of foreign atoms, such as adatoms or inatoms (defects embedded in the Pd overlayer) impact rather locally on the Fe-Fe magnetic exchange interaction. In Supplementary Figure 1a, we plot the change induced by the impurities on the substrate nearest-neighboring Fe-Fe magnetic exchange interaction for Fe atoms at the immediate vicinity of the inatoms, $\left\langle J^{\mathrm{subs}}_{\mathrm{Imp}} \right\rangle_{\mathrm{1NN}} - \left\langle J^{\mathrm{subs}}_{\mathrm{Pure}} \right\rangle_{\mathrm{1NN}}$, as function of the atomic number of the impurities. The symbol $\langle\ \rangle$ means that we proceed to an averaging over the pair of interactions since the Fe atoms surrounding the impurities are not necessarily equivalent (see Supplementary Figure 1c).

It is clearly seen that both 3d and 4d inatoms decrease the magnitude of the nearby surface atoms exchange interaction. Thus, while the substrate interestingly becomes locally less ferromagnetic, the change on the isotropic exchange follows an inverse parabolic behavior with a minimum at (or close to) half-filling. This is very similar to the shapes observed for the skyrmion-impurity binding energies shown in the main manuscript (see main manuscript Figure 2c-d). The averaged magnetic interaction, $\left\langle J^{\mathrm{inatom}} \right\rangle_{\mathrm{1NN}}$, between the inatoms and the nearest neighboring substrate atoms (see Supplementary Figure 1b) follows a similar shape as function of band filling across the periodic table with a dip at half-filling. Because of the smaller exchange splitting, the magnetic exchange interactions characterizing 4d inatoms are much smaller than those of 3d inatoms. Among the 3d impurities, Mn is the weakest coupled inatom to the substrate atoms since it lies close to the transition from antiferromagnetic to ferromagnetic coupling. Overall this is the result of the band

filling and hybridization mechanisms discussed in the main manuscript and in Supplementary Note 2.

Likewise for the inatoms, both 3d and 4d adatoms reduce locally the ferromagnetic behavior of the substrate by decreasing the exchange interaction among the neighboring Fe atoms (see Supplementary Figure 2a). The impact of the band filling is similar to what was found for the inatoms, with a local minimum for Fe among the 3d series and for Tc among the 4d series (instead of Mo among the 4d inatoms). Although the dependence on band-filling is rather similar, the magnetic exchange interactions between the adatoms and the nearest neighboring Fe substrate atoms decrease strongly when compared to the inatoms case (see Supplementary Figure 2b), which indicates that the resulting stability behavior of the skyrmions at the vicinity of the defects can be very different from what was found for inatoms. Adatoms contrary to inatoms eletronics states hybridize indirectly with those of the Fe surface atoms. In fact, the Pd overlayer atoms between the adatoms and the Fe substrate mediate the interactions , which explains the lowering of the magnitude of the interactions.

Treatment of bad Heisenberg atoms The nature of the investigated substrate and of the different impurities indicate that the extended Heisenberg model is not necessarily the ideal one since the magnetic moments carried by the Pd overlayer together with many of the 4d-inatoms have an induced nature with a strong dependence on their magnetic surrounding. For instance, the Fe atoms induce a sizable spin-moment on Pd of 0.3 μ_{B} (see main manuscript Figure 1b). One can

include the treatment of bad Heisenberg moments using a scheme based on the susceptibility of such atoms to carry a spin moment at the vicinity of “good” Heisenberg moments, mainly their nearest neighbors. We assume for example that the moment of a Pd atom is related to the nearest neighboring Fe atoms via:

$$\mathbf{M}_{\mathrm{Pd}} = \sum_{j} \chi_{\mathrm{Pd,Fe}_j} \mathbf{M}_{\mathrm{Fe}_j}. \tag{2}$$

Once the susceptibility calculated, one can include the “bad” Heisenberg moments into Supplementary Equation 1, which leads to an effective renormalization of the substrate magnetic exchange interactions as discussed in Ref. [5]. Using such a scheme, the nearest neighboring magnetic interactions among the Fe atoms of the defect-free substrate stiffens ($\left\langle \widetilde{J}^{\mathrm{subs}}_{\mathrm{Pure}} \right\rangle_{\mathrm{1NN}}$ = 19.0 meV), meaning that once a skyrmion is created, the spin moment would rotate slower when compared to the case without renormalization. Here, we neglect the renormalization induced by Ir at the vicinity of Fe since the induced magnetic moment is rather small (0.017 μ_{B}). This scheme based on the renormalization of the magnetic interactions is found to be important for the treatment of the 4d inatoms and some of the 4d adatoms (Ru, Rh, Pd) since their spin-moments vary strongly as function of the surrounding magnetic texture. Thus, these elements are treated similarly to the Pd substrate overlayer. Despite the fact that the the spin moment of Ni inatom and 4d inatoms are of the same order of magnitude, Ni spin moment is less sensitive upon rotation compared to the 4d inatoms. Therefore we treat Ni as a good Heisenberg atom. This renormalization scheme gives a better agreement with the ab initio results concerning the energetics of skyrmions.

In Supplementary Table 3, we give information regarding the averaged Fe-Fe magnetic exchange interaction, the renormalized Fe-Fe magnetic exchange interaction ($< \widetilde{J}^{\mathrm{subs}}_{\mathrm{Imp}} >_{\mathrm{1NN}}$) and the

magnetic anisotropy energy (K^{imp}) for the 3d-inatoms and 3d-adatoms. The magnetic anisotropy energy of the inatom defect (K^{imp}) are of the same order of magnitude than that of defect-free Fe substrate (K^{subs} = -0.6 meV). We also provide a similar table for the 4d-inatoms and 4d-adatoms Supplementary Table 4. However, we skip the magnetic anisotropy energy for 4d-inatoms and for the late 4d-adatoms since these elements behave like Pd.

Skyrmion-defects binding energies Using this extended Heisenberg model with parameters extracted from ab initio, we investigate the binding energy of a single skyrmion at the vicinity of 3d and 4d impurities similarly to what was done from full ab initio simulations. The relaxed magnetic textures, with zero magnetic torque acting on each magnetic moment, were computed by minimizing the Heisenberg Hamiltonian with respect to the spherical angles (θ_i, ϕ_i), defining the orientation of every magnetic moment $\vec{e}_i = (\cos\phi_i \sin\theta_i,\ \sin\phi_i \sin\theta_i,\ \cos\phi_i)$.

The binding energies calculated from the full ab initio approach are in qualitative good agreement with those obtained with the extended Heisenberg model, as can be seen in the main manuscript Figure 2c-d and Supplementary Figure 3a-b, respectively. The general shape of the energy profiles are the same, however, the magnitude and the interaction nature of some impurities can be different. Once more, this is due to the assumption inherent to the Heisenberg model, where the magnitude of the moments is assumed to be unchanged upon rotation. This aspect cannot be totally curred with the renormalization scheme described previously.

Even with such a disadvantage, the model has the merit to provide a detailed understanding of

the different contributions of the tensor of magnetic interactions to the calculated binding energies. These contributions are plotted as function of the atomic number in Supplementary Figure 4a-b. For both the 3d and 4d inatoms, the main energy contribution comes from the exchange energy (blue line), which settles the tendency of the impurities to pin or repel. The impact of the DMI, the magnetic anisotropy and the symmetric exchange energies on the binding energy is rather minor (green, purple and orange lines, respectively). In general, the exchange interaction disfavors the skyrmion particle being close to a 3d inatom in contrast to 4d inatoms. This is in line with the discussions made in the main text, where we related the trends of the binding energies to the hybridization processes impacting on the magnitude and nature of the direct exchange interactions.

From our analysis, we found that there are two counter-acting contributions to the exchange energy: (i) one comes from the substrate, which is locally lowered by the presence of the impurities and thus favors the rotation of the magnetic moments created by the skyrmions, i.e. favors pinning (see red line in Supplementary Figure 4c); (ii) the other comes from the impurity-substrate interactions, which stiffens the magnetic complex, impuriy-substrate, and disfavors the creation of skyrmions, i.e. leads to repulsion (see black line in Supplementary Figure 4c). Thus, for 3d inatoms, the contribution due to impurity exchange interaction prevails over the reduction of the exchange energy occurring in the Fe substrate, which lead to repulsion. In contrast, since the moments of 4d inatoms are induced by the surrounding Fe substrate atoms, they only contribute to a moderate renormalization of the Fe substrate exchange energy. In other words, pinning is favored (blue curve in Supplementary Figure 4b).

Supplementary Note 2 − Electronic structure of inatoms at the vicinity of skyrmions

As discussed in the main manuscript for V and Tc inatoms, i.e. impurities embedded in Pd deposited on Fe/Ir(111), the mechanism behind the impurity-skyrmion stabilization for inatoms is connected to the hybridization between the electronic states of the inatoms and of the Fe substrate. Thus, the skyrmion stability at the vicinity of an inatom can be related to the electronic structure in a transparent way. The electronic structure impacts on the nature of the magnetic interactions, which as discussed in the previous section defines the magnitude and sign of impurity-skyrmion binding energies. Indeed, due to this hybridization the virtual bound state (VBS) splits into bonding and anti-bonding states, which depending on their filling impact on the stability of a given configuration. For the adatoms, hybridization with the Fe substrate is mediated via the intermediate Pd atoms. The band filling effect is observed for the adatoms as well and could be explained in terms of the extended Heisenberg model. However, a direct link to the electronic structure features is not as transparent as it is for the inatoms. In the following, we discuss the local density of states (LDOS) of the inatoms not shown in the main manuscript (Cr, Mn, Fe, Co, Ni, Nb, Mo, Ru, Rh).

When considering collinear magnetism, early (late) 3d and 4d inatoms are found to couple antiferromagnetically (ferromagnetically) to the Fe substrate (see main manuscript Figure 1b). The majority-spin VBS of V, Cr and the minority-spin VBS of Fe, Co and Ni hybridize with the minority-spin states of Fe substrate giving rise to a band with large width carrying clear bonding and anti-bonding states (see lower panel in Supplementary Figure 5a-f, red curve). The majority-spin bands of the Fe substrate atoms are almost filled, providing a low amount of unoccupied

states available for hybridization with the minority-spin states of V, Cr and Mn, which explains the sharpness of their VBS. Moving the 3d-impurities towards the skyrmion core leads to a broadening of the bonding states, which were initially occupied for most of the investigated elements. The non-collinearity of the magnetic moments open additional hybridization channels, leading to the intensity reduction of this bonding-state, which is a process unfavorable energetically explaining the repulsive nature of the 3d-inatoms.

Mn is a special case since it lies close to the antiferromagnetic-ferromagnetic transition point, with a rather weak magnetic coupling to the Fe substrate (see previous section), as shown in Supplementary Figure 5c. In this particular case, other mechanisms can play a more important role in stabilizing the skyrmion at the vicinity of the impurity. For instance, one notices that the majority-spin band center of the Fe substrate experiences a shift to lower energies in the non-collinear case, which leads to a gain in energy.

As mentioned in the main text, 4d inatoms tend to pin the skyrmions (see main manuscript Figure 2d). The smaller exchange splitting of the d-states compared to the one of the 3d-inatoms has a tremendous impact on the electronic structure and thus on the investigated energy profiles. In particular, the minority-spin VBS for early 4d inatoms, and majority-spin VBS, for late 4d inatoms are not inert as the ones of the 3d-series since they are located close to the Fermi energy (see Supplementary Figure 6a-e, red curve). Therefore, moving the 4d-inatoms towards the core of the skyrmion leads to additional hybridization broadening of the VBS with the creation of bonding and anti-bonding states (see Supplementary Figure 6g-k, grey curve). Here, interestingly, the

bonding states get occupied stabilizing thereby the non-collinear spin-texture at the vicinity of the 4d inatoms.

Supplementary References

1. Ebert, H. & Mankovsky, S. Anisotropic exchange coupling in diluted magnetic semiconductors: ab initio spin-density functional theory. *Phys. Rev. B* **79**, 045209 (2009).

2. Liechtenstein, A., Katsnelson, M., Antropov, V. & Gubanov, V. Local spin density functional approach to the theory of exchange interactions in ferromagnetic metals and alloys. *Journal of Magnetism and Magnetic Materials* **67**, 65 – 74 (1987).

3. Lounis, S. & Dederichs, P. Mapping the magnetic exchange interactions from first principles: Anisotropy anomaly and application to Fe, Ni, and Co. *Phys. Rev. B* **82**, 180404 (2010).

4. Dupé, B., Hoffmann, M., Paillard, C. & Heinze, S. Tailoring magnetic skyrmions in ultra-thin transition metal films. *Nature Commun.* **5**, 4030 (2014).

5. Polesya, S. *et al.* Finite-temperature magnetism of Fe_xPd_{1-x} and Co_xPt_{1-x} alloys. *Phys. Rev. B* **82**, 214409 (2010).
